# Ecological Risk Assessment and Source Identification of Potential Toxic Elements in Farmland Soil of Nanyang Basin, China

**DOI:** 10.3390/toxics13050342

**Published:** 2025-04-25

**Authors:** Weichun He, Xiaowei Fei, Hao Guo, Guangyu Zhang, Mengzhen Li, Yuling Jiang

**Affiliations:** 1 School of Geographic Sciences, Xinyang Normal University, Xinyang 464000, China; heweichun@xynu.edu.cn (W.H.); 54838@xynu.edu.cn (X.F.); guohao@xynu.edu.cn (H.G.); zhangguangyu@xynu.edu.cn (G.Z.); 0509@xynu.edu.cn (M.L.); 2 Henan Key Laboratory for Synergistic Prevention of Water and Soil Environmental Pollution, Xinyang Normal University, Xinyang 464000, China

**Keywords:** potential toxic elements, risk assessment, spatial distribution, source identification

## Abstract

This study investigated spatial distribution features and ecological risks of eight potential toxic elements (Cr, Ni, Cu, Zn, Pb, As, Cd, and Hg) in surface soil samples (0–20 cm) collected from farmland in the Nanyang Basin, China. This research also aimed to analyze the sources of these elements. Its findings revealed that the mean contents of Cr, Ni, Cu, Zn, Pb, As, Cd, and Hg were 54.35, 26.57, 25.20, 82.09, 22.17, 8.27, 0.17, and 0.13 mg·kg^−1^, respectively, all of which were lower than their corresponding risk screening values. However, the mean contents of Cu, Zn, Cd, and Hg exceeded the background values of Henan Province. Spatial distribution analysis revealed that Cr and Ni exhibited similar patterns, with high contents primarily observed in the western part of the research area. Generally speaking, Cu, Zn, and Pb contents were higher in the south and lower in the north, whereas Hg, As, and Cd displayed a scattered distribution of high-value areas. Ecological risk assessment indicated that Hg and Cd posed relatively high risks, with their comprehensive ecological risk indexes (*RI*s) predominantly classified as moderate. Source identification suggested that As primarily originates from agriculture, Cd from industry sources, Hg from coal combustion, and the remaining elements from mixed sources, including parent material, transportation, and agriculture.

## 1. Introduction

Soil is fundamental to human survival and critical for sustainable agricultural development in China [1]. With the fast growth of China’s population and the accelerated development of urban areas and industries, soil pollution has emerged as a critical issue [2,3]. Over the years, human activities have introduced a wide range of organic and inorganic pollutants, such as pesticides, fertilizers, industrial waste, domestic garbage, and automobile emissions, into the soil [4,5,6], altering the natural background values of soil chemical elements in urban and surrounding areas and significantly increasing the content of soil potential toxic elements (PTEs) [7]. As a result, soil quality has declined, threatening both crop production and human health [8,9,10]. Recent national assessments highlight the severity of this issue. According to data from the Ministry of Land and Resources and the Ministry of Environmental Protection, it is indicated that 19.4% of cultivated land samples exceed the permissible thresholds for PTEs. In light of these findings, a comprehensive investigation into PTEs’ spatial distribution, ecological risks, and sources in farmland soils is imperative. Such research is vital for devising effective pollution-control strategies, enhancing agricultural soil quality, and ensuring food security and public health.

Presently, there exist a number of approaches for accessing soil PTE ecological risk. Among them, the potential ecological risk index approach is broadly applied because it can not only assess the ecological hazard of individual PTEs but also provide an understanding of the combined effects of multiple PTEs. Source apportionment methods for PTEs in soils are also relatively well established. The positive matrix factorization (PMF) model has become one of the most popular receptor models, and it has been successfully utilized in determining PTE pollution sources in agricultural soils because it can handle missing and imprecise data without requiring the determination of complex source profiles [11,12]. Through PMF analysis of farmland soils in the Nanyang Basin, the sources of PTEs can be effectively distinguished [13] and pollution sources can be determined [14].

Nanyang is situated in Henan Province’s southwestern part and recognized as the largest agricultural city in the province. It is often referred to as the “Granary of Zhongzhou”. With its abundant agricultural resources and strategic geographical advantages, Nanyang serves as a major production hub for grain, oil, and tobacco in China and is designated as a national commodity grain base. Its agricultural development plays a crucial role not only in Henan Province but also at the national level. In addition to diverse agricultural products, the region is rich in mineral resources [15]. The primary food crops cultivated in Nanyang include wheat, corn, and rice. To further enhance agricultural production efficiency and quality, Henan Province has established a 1500-acre, high-standard farmland demonstration zone in the Nanyang Basin, aiming to consolidate the important position of the Nanyang Basin in national agriculture. The Nanyang Basin, a densely populated agricultural region, faces a significant challenge from PTE pollution in its farmland soils. This pollution threatens agricultural productivity and poses a direct risk to local residents’ health. Reports of excessive PTE levels in certain areas [16,17] underscore the urgency of this issue. Therefore, a systematic investigation into the contamination levels, spatial distribution, and contamination sources of soil PTEs is critically needed to provide a scientific basis for valid soil contamination prevention, control, and remediation strategies. This study focuses on the farmland soils of the Nanyang Basin as the research subject, quantifies the PTE contents within the soil, assesses the ecological risk using the potential ecological risk index, identifies PTE pollution’s spatial distribution patterns in farmland soils of the Nanyang Basin, and determines the sources of pollution by integrating the PMF model. The findings of our work aim to inform regional land use planning, guide agricultural structural adjustments, and uphold the development of effective pollution management policies.

## 2. Materials and Methods

### 2.1. Overview of the Study Area

Nanyang is situated in the southwestern area of Henan Province, at the intersection of Hubei, Henan, and Shanxi Provinces. This area spans the longitudes 110°58′ E to 113°49′ E and the latitudes 32°17′ N to 33°48′ N. The region is characterized by a basin topography, enclosed by mountains on three sides and open to the south, positioned along the Qinling Mountains–Huaihe River Line. The average altitude is about 150 m a.s.l [18]. Winters are cold and dry, while summers are hot and humid. Therefore, the region is rich in mineral resources, with significant deposits of gold, pyrite, lead–zinc, silver, copper, and phosphate. The average annual temperature is about 16.8 °C [19]. The annual precipitation ranges between 750 and 850 mm [18]. According to the results of our experiments, the pH of the soil in the research area ranges from 5.23 to 7.96, with the majority of soils being acidic (pH < 7). The soil organic matter (SOM) contents vary between 14.79 and 50.94 g/kg. The mean content is 30.45 g/kg. The range of cation exchange capacity (CEC) contents is 14.67 to 44.36. The average content is 24.01 cmol (+)/kg, which means that the soil is medium-fertility soil. The region’s main agricultural products are peanuts, wheat, corn, and rice, while the region is also renowned for its livestock and sericulture industries. Notably, Nanyang yellow cattle, black pig, and tussah production hold national recognition, with Nanyang yellow cattle ranked as the premier breed among China’s five major cattle breeds.

### 2.2. Sample Collection and Testing

**Figure 1:**
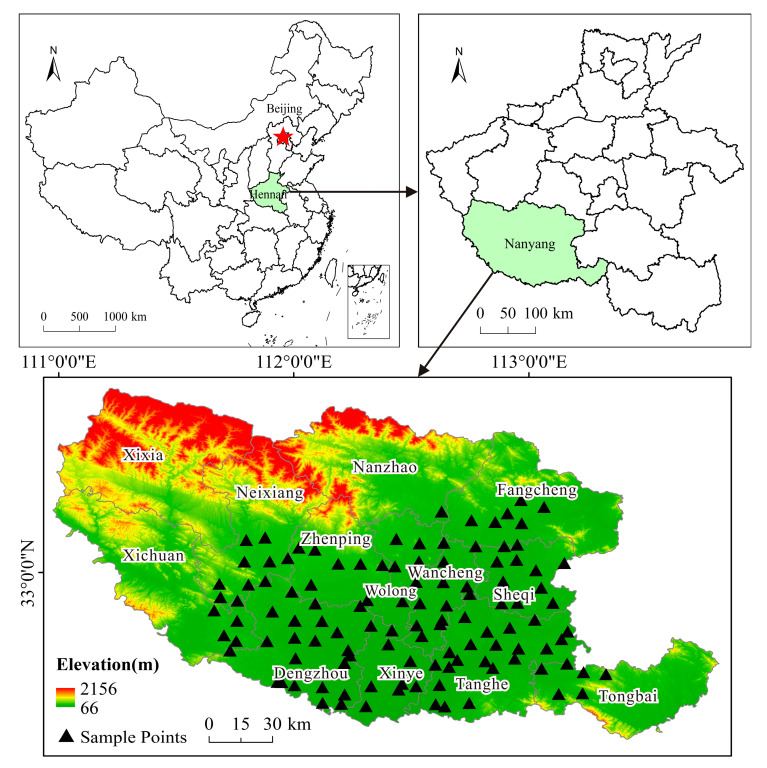
Spatial distribution of sampling points in research area.

Soil samples were gathered near grid points’ centers by using a uniformly arranged point method with a grid size of 10 km × 10 km. In order to minimize potential contamination from anthropogenic sources, specific buffer zones were established around sampling locations. Sampling points were situated at distances greater than 2 km away from cities, towns, residential regions, major traffic routes, and industrialized enterprises. Furthermore, a buffer of at least 1 km was maintained from villages, and a minimum distance of 200 m was observed from roads and ditches in the farmland. Following these criteria, in total, 126 samples from farmland soils were collected within the research area (Figure 1).

At the sampling points, surface soil samples (0–20 cm) were gathered using a “plum blossom” sampling pattern. Within each 1 km^2^ grid, a composite sample was gained by collecting multiple subsamples from 3 to 5 locations within a 100 m radius around the central sampling point, which were then thoroughly mixed. At every sampling point, approximately 1 kg of surface soil (0–20 cm) was collected. During the sampling process, plant residues, brick and tile fragments, gravel, and other debris on the soil surface were all removed. The soil specimens were put in cloth bags and labeled with sample numbers. Concurrently, the study recorded the geographical coordinates of the sampling points, current land use, and surrounding environmental information.

In the laboratory, the specimens were air-dried naturally and sieved via a 10-mesh (1.7 mm) nylon sieve before pH and cation exchange capacity (CEC) determination. For detecting PTEs and organic matter (OM), the study randomly collected approximately 25 g of soil from 30 points within the 10-mesh sample and sieved the soil via a 100-mesh nylon sieve with an aperture of 0.15 mm.

Soil specimens were digested using the HNO_3_-HF-HClO_4_ system to determine elements’ contents, including Ni, Cd, Cu, Pb, Zn, and Cr, as per the Technical Specification for Soil Environmental Monitoring (HJ/T 166-2004) [20]. These contents were measured by utilizing an inductively coupled plasma mass spectrometer (XSeries-2 ICP-MS, Thermo Fisher Scientific, 81 Wyman Street, USA) as specified by Thermo Fisher (HJ 766-2015) [21]. After aqua regia digestion, As and Hg contents were analyzed by an atomic fluorescence spectrophotometer (AFS-3100, Beijing Haiguang Instrument Co., LTD, Beijing Shunyi) in accordance with standards GB/T 22105.1-2008 [22] and GB/T 22105.2-2008 [23]. All reagents used in the tests were of analytical-grade purity, with deionized water employed throughout procedures. Quality control measures were implemented for each batch of samples, including parallel tests, blank tests, and standard recovery tests, using GSS-2 as the standard reference soil sample. For guaranteeing the analytical results’ reliability and accuracy, every batch included three standard specimens and three blank specimens. Parallel specimens constituted 20% of the total batch. Recovery rates for the analyzed PTEs ranged between 85% and 108%. Phase matching errors for parallel samples were maintained between 5% and 25%.

### 2.3. Data Analysis

#### 2.3.1. Assessment of PTE Pollution

For assessing PTEs’ contamination levels, the single pollution index (*PI*), ecological risks of a single potential toxic element (*E_i_*), and the comprehensive ecological risk index (*RI*) were employed [24,25,26]. The potential ecological risk index (*RI*) is a significant indicator for assessing soil PTEs’ risk levels. It quantifies both the risk levels of individual elements and their cumulative impact on the environment. The *PI*, *E_i_*, and *RI* indexes are calculated as follows:

(1) $$\begin{array}{c} PI=\frac{C_{i}}{C_{n}^{i}}\\ E_{i}=\sum_{i=1}^{n}\left(T_{i}\times PI\right)\\ RI=\sum_{i=1}^{n}E_{i} \end{array}$$

In the formulae, *PI* represents the single pollution index of a PTE in soil, *E_i_* stands for the potential ecological risk of the *i*-th PTE, *RI* represents the comprehensive potential ecological risk index of a PTE in soil, *C_i_* means the content of the *i*-th PTE (mg·kg^−1^), $C_{n}^{i}$ indicates the background value of the *i*-th PTE, and *T_i_* represents the toxicity coefficient of the *i*-th PTE, as depicted in Table 1 [24]. Categories for potential ecological risk indexes refer to Table 2 [24].

#### 2.3.2. Correlation Analysis

Correlation analysis means examination of the relationship between two or more factors to determine how they are related. It determines the association degree among variables. In the natural sciences, the Pearson correlation coefficient is broadly applied in view of its broad applicability and high reliability. It is particularly effective for analyzing complex data influenced by multiple factors [27]. The Pearson correlation coefficient was utilized here to study PTEs’ correlations. The Pearson correlation coefficient is defined as the quotient of covariance and the standard deviation of estimated samples. Its mathematical expression is as below:

(2) $$r=\frac{\sum_{i=1}^{n}\left(x_{i}-\bar{x}\right)\left(y_{i}-\bar{y}\right)}{\sqrt{\sum_{i=1}^{n}{\left(x_{i}-\bar{x}\right)}^{2}\sum_{i=1}^{n}{\left(y_{i}-\bar{y}\right)}^{2}}}$$

In the formula, $\bar{x}$ and $\bar{y}$ stand for the means of *n* trial values. The value of *r* ranges from −1 to 1, with higher absolute values implying superior dependency among variables [28].

#### 2.3.3. Positive Matrix Factorization (PMF) Model

The positive matrix factorization (PMF) model is a multivariate factor analysis tool originally proposed by Paatero (1994) for source assignment of contaminants in the environment. The fundamental principle is to decompose the receptor’s original data matrix (X) into a factor score matrix (G), a factor loading matrix (F), and a residual matrix (E) by using the least-squares method of minimum iteration [29,30,31]. This method is useful in identifying the number of PTE sources and determining the contributions of various sources to PTE accumulation and can better resolve the sources of pollutants [32]. The formula is as below:

(3) $$X_{ij}=\sum_{k=1}^{p}\left(G_{ik}\times F_{kj}\right)+E_{ij}$$

In the formula, *X_ij_* represents the content of the *j*-th element in the *i*-th sample, *p* means the number of factors, *G_ik_* means the relative contribution of the *k*-th source in the *i*-th sample, *F_kj_* indicates the eigenvalue of the *k*-th source for the content of the *j*-th PTE, and *E_ij_* stands for the residual of the *j*-th element in the *i*-th sample. The original matrix, *X*, is decomposed through the PMF model to obtain the optimal matrices *G* and *F*, minimizing the objective function, *Q* [33], thereby addressing the dimensionless parameter issue. *Q* is defined as below:

(4) $$Q={\sum_{i=1}^{n}\sum_{j=1}^{m}\left(\frac{E_{ij}}{U_{ij}}\right)}^{2}$$

The formula for calculating the uncertainty, *U_ij_*, is as follows:

(1) When the content ≤ method detection limit (MDL):

(5) $$U_{ij}=\frac{5}{6}\times MDL$$

(2) When the content > MDL:

(6) $$U_{ij}=\sqrt{{\left(\sigma \times X_{ij}\right)}^{2}+MDL^{2}}$$

In the formula, *U_ij_* represents the uncertainty, σ indicates the standard deviation, *X_ij_* means the elemental content (mg·kg⁻^1^), and MDL means the method detection limit (mg·kg⁻^1^). The detection limits for As, Cd, Cr, Cu, Hg, Ni, Pb, and Zn are 2, 0.05, 3, 2, 0.005, 5, 2, and 3 mg·kg⁻^1^, respectively [34].

### 2.4. Statistical Tools

Statistical analysis of descriptive data, raw data processing, and pollution index calculations were conducted utilizing Microsoft Excel 2021. ArcGIS 10.2 was employed for mapping and spatial analysis. SPSS 27.0 and Origin 2021 were employed to perform correlation analysis [35]. Additionally, primary sources of PTEs in soil of the Nanyang Basin were identified, with their respective contribution rates quantified, utilizing EPA PMF 5.0 software.

## 3. Results and Discussion

### 3.1. Statistics of PTE Contents in Soil

This study conducted a descriptive statistical analysis of various PTEs of agricultural soils in the Nanyang Basin, and the results are shown in Table 3. The mean contents of Cu, Zn, Cd, and Hg exceeded the background values of soil in Henan Province [4], but the average contents of all PTEs failed to exceed the national risk screening values (GB 15618-2018) [36]. Regarding the range of variation among different PTEs, excluding maximum values for Cu, Zn, and Cd higher than risk screening values, the maximum values for the other elements were all lower than the screening values. The spatial coefficients of variation (CVs) of PTEs in the study region took the following order: Hg > Cd > As > Zn > Cu > Ni > Pb > Cr. Compared with the risk screening values of agricultural land in GB 15618-2018 [36], Cu exhibited the highest exceedance rate at 10.32%, followed by Zn and Cd, both at 4.76%, while the remaining elements showed no exceedance. These exceedance rates suggest that Cu, Zn, and Cd contents showed varying degrees of contamination in the samples.

### 3.2. Spatial Distribution of PTEs in Soil

**Figure 2:**
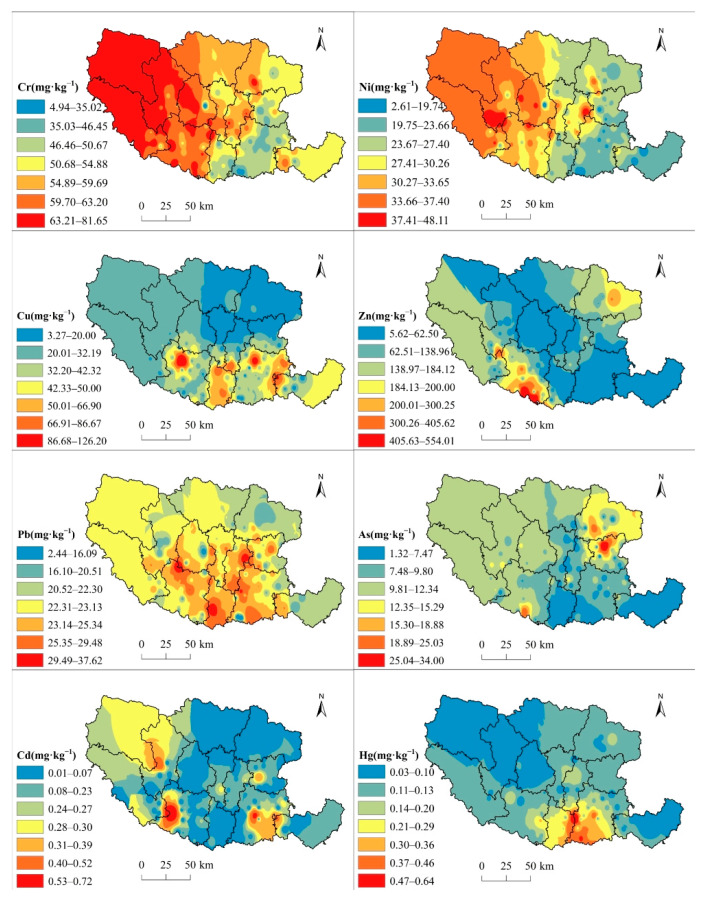
Spatial distribution of PTEs.

The spatial distribution of the eight PTEs (Figure 2) was analyzed utilizing inverse-distance-weighted (IDW) interpolation in ArcGIS. The Cr and Ni spatial distribution patterns were similar, with high contents clustered in the western part of the research area and low contents mainly distributed in the east. Cu, Zn, and Pb also exhibited analogous spatial distribution patterns. Generally, their contents were high in the south and low in the north. Notably, Cu contents varied considerably between the northern and southern regions, with the southern region exhibiting significantly higher levels. The pollution was present in varying degrees in the northern part of Dengzhou, the central part of Xinye, and the northwestern and eastern parts of Tanghe. Zn contents were relatively high in southern Dengzhou and eastern Fangcheng, suggesting potential contamination, whereas other areas had lower Zn levels, posing minimal risk. Pb contents were highest in the central and southern regions of the research area and gradually decreased toward the periphery.

The spatial distribution of As is generally similar to that of Zn, but compared with Zn, the high-value area of As in the southwest has shrunk, while that in the northeast has expanded. The distribution of high-value areas for Cd is relatively decentralized, particularly in the western part of Dengzhou, western Neixiang, and central to eastern Tanghe, indicating varying degrees of pollution, which may be linked to industrial activities. In contrast, the remaining areas show relatively low Cd contents. Hg forms high-value areas in the southwest of Tanghe, the south of Wancheng, and the east of Xinye, with a certain degree of pollution. The Hg content is relatively low in the other areas.

### 3.3. Potential Ecological Risk Assessment

PTEs’ potential ecological risk statuses in farmland soils in the Nanyang Basin were assessed using the potential ecological risk index (*E_i_*) and the comprehensive ecological risk index (*RI*) (Table 4). This assessment indicated that Cr, Ni, Cu, Zn, Pb, and As posed a low ecological risk. However, Hg posed a significant high ecological risk in farmland soils of the Nanyang Basin. Specifically, a substantial proportion of sampling points were classified as having considerable to extremely high risk: 8.73% were at extremely high risk, 30.95% at high risk, and 53.97% at considerable risk. A small fraction of sampling points presented moderate (5.56%) or low (0.79%) risk. Thus, Hg was identified as the primary ecological risk factor in the farmland soils of the Nanyang Basin. Cd was the second most concerning element. A small percentage of sampling points were classified as extremely high (0.79%) or high risk (3.17%) for Cd, 19.84% were at considerable risk, 69.05% at moderate risk, and 7.14% at low risk. The contents of Hg and Cd exceeded Henan Province’s background values, and their higher ecological risks were closely related to local anthropogenic activities like industrial and coal combustion emissions. Attention should be given to contamination with Hg and Cd.

From the *RI* value for each soil sampling point, the *RI* risk level was derived (Table 2) and the proportion of all soil samples of the same class to the total soil sample points was calculated to obtain the percentage of *RI* risk level, as shown in the last column of Table 4. The average *RI* was 280.51, with values ranging from 86.19 to 944.84, indicating an overall moderate ecological risk. In terms of the proportion of sampling points at different *RI* risk levels, 2.38% of the points were at low risk, 74.60% at moderate risk, 19.84% at considerable risk, and 3.17% at high risk. Additionally, extensive application of chemical fertilizers and pesticides in agricultural practices may have propelled PTE accumulation in farmland soils, further elevating ecological risks.

### 3.4. Source Identification of PTEs

#### 3.4.1. Correlation Analysis of PTEs

**Figure 3:**
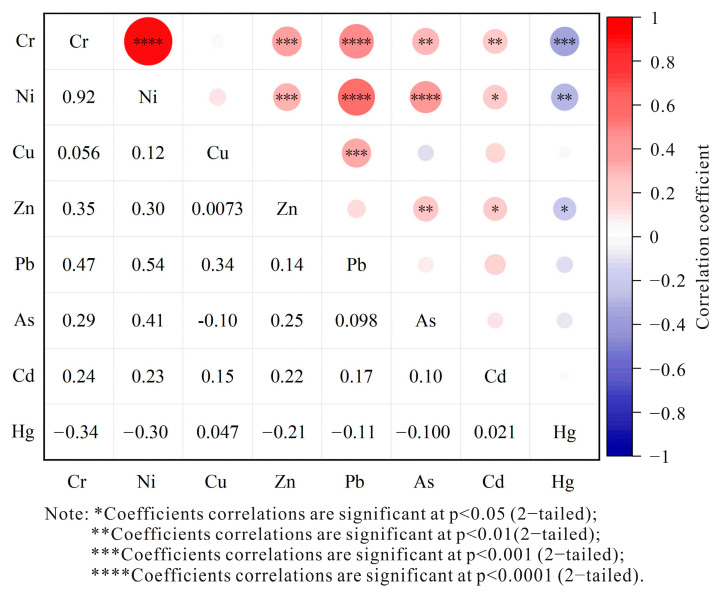
Correlation analysis of PTEs in farmland soils of Nanyang Basin.

Correlation analysis is a preliminary approach to identifying PTEs that share a common source, laying the groundwork for subsequent pollution source apportionment. By statistically analyzing the relationships among PTEs, their homogeneity can be assessed [37]. Previous studies suggested that a significant correlation among PTEs in soil may indicate a common or combined pollution source, whereas a lack of correlation suggests multiple independent sources [38,39]. Therefore, correlation analysis is a critical step in determining the origins of PTE contamination [40]. Figure 3 presents the Pearson correlation analysis of PTEs in the soil, created using Origin 2021 software. The analysis exhibited a powerful positive correlation between Ni and Cr (*r* = 0.92), indicating significant homogeneity and a likely shared source. The correlation coefficients for Pb-Cr, Pb-Ni, Pb-Cu, Zn-Cr, and Zn-Ni ranged from 0.30 to 0.54, suggesting low to moderate correlations, which implies that these PTEs may originate from a common or combined pollution source and exhibit interdependent behavior. In contrast, the correlations between As, Cd, and Hg and other PTEs were not significant. The correlation coefficients between Cd and Hg and other PTEs were all below 0.30, indicating that Cd and Hg homogeneity with respect to other PTEs is weak, and the accumulated pollution with these elements may come from separate pollution sources.

#### 3.4.2. Source Identification of PTEs Based on PMF

**Figure 4:**
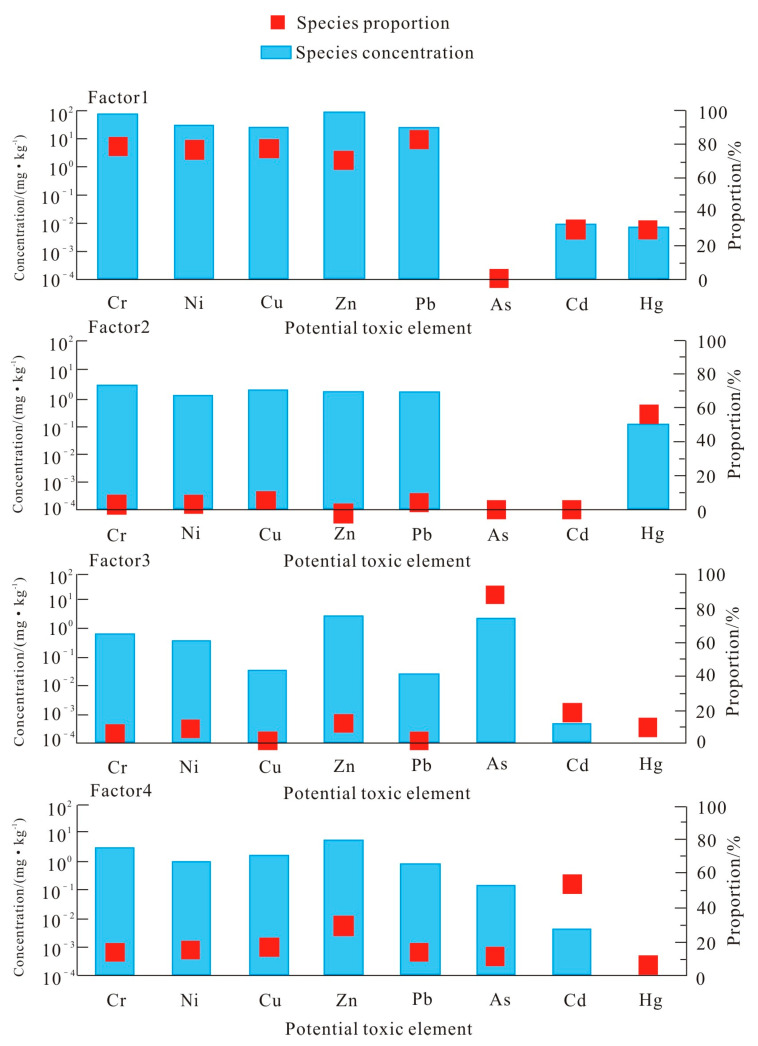
Source identification of soil PTEs based on PMF.

The EPA PMF 5.0 model was employed to quantitatively analyze PTE pollution sources in farmland soils of the Nanyang Basin. The contribution proportions of PTEs of each factor are shown in Figure 4. For determining the optimal number of factors, this study conducted 20 runs, randomly selecting the initial points for three to six factors. After the calculation, a stable ratio of Q_Robust_ to Q_True_ was obtained while the number of factors was 4, achieving the best model fitting effect. The residuals of all PTE contents were within the range of −3 to 3 [41], except for the R^2^ values for Zn and Cu fitting, which were, respectively, 0.42 and 0.48; the R^2^ values of the other elements were higher than 0.6; the R^2^ value of Cr fitting was higher than 0.6; the R^2^ values of Ni and Pb fitting were higher than 0.7; and the R^2^ values of As, Cd, and Hg fitting were higher than 0.99. These results suggest that the PMF model developed in this study exhibits robust overall fitting performance and effectively elucidates the pollution sources of PTEs in farmland soils of the Nanyang Basin.

Factor 1 represents a complex combination of PTEs, with the highest contribution rates observed for Pb (80.1%), Cr (77.1%), Cu (76.6%), Ni (74.7%), and Zn (69.4%). The strong correlation between Cr and Ni (r = 0.92), along with similar spatial distribution patterns (Figure 2), suggests a common external pollution source. According to Table 1, the Cr and Ni mean contents do not exceed Henan Province’s background soil values, indicating that such elements are less affected by anthropogenic activities. This finding aligns with previous research, which suggests that natural weathering of soil and bedrock is Ni’s and Cr’s main source [42,43,44]. At the same time, Factor 1 shows a high contribution of Pb, Zn, and Cu. The sources of Cu and Zn are relatively widespread.

Utilizing pesticides and fertilizers in agriculture is a contributor to Cu and Zn enrichment in soil. Furthermore, the well-developed livestock and poultry industry in the research area is another source of these elements. Specifically, the excretion of animal manure results in organic fertilizers with elevated Cu and Zn contents, which are subsequently applied to farmland, further contributing to soil enrichment. Several studies have also identified Pb, Cu, and Zn as indicative elements of traffic-related activities, primarily originating from leaded gasoline combustion, engine wear, vehicle component degradation, and tire friction [45,46]. Pb has a certain correlation with Cr and Ni, suggesting that Pb, Cr, and Ni may come from a common pollution source. Therefore, it is comprehensively indicated that Factor 1 is a composite source comprising natural parent material, traffic emissions, and agricultural activities.

The high loading element of Factor 2 is Hg, accounting for 54.8%. The Hg content in the research area, on average, is 4.33 times superior than Henan Province’s background value, indicating a certain degree of accumulation. Hg is widely recognized as a byproduct of coal and fossil fuel combustion [47,48,49]. During the combustion process, Hg turns into a gaseous state and enters the atmosphere and can be deposited in farmland soil through atmospheric deposition and cause pollution [50]. Based on these findings, Factor 2 is identified as being primarily associated with the atmospheric deposition and emissions of coal combustion.

Factor 3 is mainly characterized by As, accounting for 88.4% of its variance. As exhibits a relatively low correlation with respect to other elements, suggesting a distinct source. Existing research indicates that agricultural production activities are significant contributors to As accumulation in soil, potentially explaining its prominence in Factor 3 [51,52]. The use of pesticides, livestock manure, and various fertilizers has been shown to contribute significantly to elevated As contents [53,54]. Additionally, industrial waste mismanagement can introduce As into farmland soil through chemical fertilizer contamination, waste accumulation, and sewage irrigation. From these findings, Factor 3 is recognized as stemming from agricultural activities. Factor 4 is primarily characterized by a high contribution of Cd, which accounts for 52.7% of this factor. Cd is a key indicator of industrial pollution, primarily influenced by industrial activities like lead–zinc mining and smelting [55,56]. The research area is abundant in mineral resources, like gold, pyrite, lead–zinc, silver, copper, and phosphate. During the ore smelting process, the release of dust-laden flue gas and the surface and underground runoff of wastewater contribute to Cd accumulation in the soil. Based on these findings, Factor 4 is identified as an industrial pollution source.

To sum up, PTE pollution’s four primary sources have been identified in farmland soils of the research region: industrial sources, coal combustion and atmospheric deposition sources, agricultural sources, and comprehensive sources (including parent material, agricultural source, and traffic sources). As mainly comes from agricultural input pollution, Cd mainly from industrial emissions, and Hg mainly from coal combustion and atmospheric deposition. Pb, Zn, Cu, Ni, and Cr are primarily affected by comprehensive sources, including natural parent material weathering, agricultural inputs, and traffic-related emissions.

## 4. Conclusions

Mean contents of Cr, Ni, Cu, Zn, Pb, As, Cd, and Hg in the soil were below risk screening values in GB 15618-2018. However, average contents of Cu, Zn, Cd, and Hg exceeded Henan Province’s soil background values. Spatial distribution analysis revealed that Cr and Ni exhibited similar patterns. Their high contents were primarily observed in the western part of the research region. Generally speaking, Cu, Zn, and Pb contents were higher in the south and lower in the north, whereas Hg, As, and Cd displayed a scattered distribution of high-value areas. Ecological risk assessment indicated that Hg and Cd posed relatively high risks, with the comprehensive ecological risk indexes predominantly classified as moderate. It is essential to strengthen soil ecological risk control in the research area, especially the control of Hg and Cd. Source identification suggested that As primarily originates from agriculture, Cd from industry sources, Hg from coal combustion, and the remaining elements from mixed sources, including parent material, transportation, and agriculture.

## Figures and Tables

**Table 1:** Toxicity coefficients (*T_i_*s) of PTEs.

Project	As	Hg	Cd	Cr	Pb	Zn	Ni	Cu
*T_i_*	10	40	30	2	5	1	5	5

**Table 2:** Classification of PTE indexes.

*E*_*i*_	Risk Level	*RI*	Risk Level
*E_i_* < 40	Low risk	*RI* < 150	Low risk
40 ≤ *E_i_* < 80	Moderate risk	150 ≤ *RI* < 300	Moderate risk
80 ≤ *E_i_* < 160	Considerable risk	300 ≤ *RI* < 600	Considerable risk
160 ≤ *E_i_* < 320	High risk	*RI* ≥ 600	High risk
*E_i_* ≥ 320	Extremely high risk	-	-

**Table 3:** Statistics of PTE contents in surface soil of research area.

PTE	Content Range	Median /(mg·kg^−1^)	Mean /(mg·kg^−1^)	Standard Deviation /(mg·kg^−1^)	CV (%)	Background Value [4] /(mg·kg^−1^)	Screening Value [36] /(mg·kg^−1^)	Exceeded Screening (%)
Cr	3.00–74.80	55.40	54.35	11.46	21.09	63.20	150	0.00
Ni	2.59–48.11	25.84	26.57	7.25	27.29	27.40	70	0.00
Cu	2.00–55.91	24.70	25.20	8.14	32.32	20.00	50	10.32
Zn	3.00–203.49	74.81	82.09	36.80	44.83	62.50	200	4.76
Pb	2.00–37.62	21.85	22.17	4.68	21.12	22.30	90	0.00
As	0.71–34.02	8.18	8.27	4.11	49.73	9.80	40	0.00
Cd	0.01–0.82	0.15	0.17	0.10	56.25	0.07	0.3	4.76
Hg	0.01–0.64	0.11	0.13	0.09	65.13	0.03	1.8	0.00

**Table 4:** Summary of statistical values pertaining to proportions of soil specimens at various degrees of potential ecological risk.

Elements	*E_i_*								*RI*
	Cr	Ni	Cu	Zn	Pb	As	Cd	Hg	
Range	0.15–2.71	0.47–8.78	0.79–33.33	0.09–8.87	0.53–8.43	0.72–34.71	5.22–349.69	35.84–856.13	86.19–944.84
Mean	1.75	4.88	7.44	1.53	5.01	8.49	73.57	177.85	280.51
Standard deviation	0.38	1.32	4.78	1.23	1.05	4.20	41.15	114.96	122.45
Low risk (%)	100	100	100	100	100	100	7.14	0.79	2.38
Moderate risk (%)	0	0	0	0	0	0	69.05	5.56	74.60
Considerable risk (%)	0	0	0	0	0	0	19.84	53.97	19.84
High risk (%)	0	0	0	0	0	0	3.17	30.95	3.17
Extremely high risk (%)	0	0	0	0	0	0	0.79	8.73	—

## Data Availability

Data are contained within the article.
